# MOntelukast as a potential CHondroprotective treatment following Anterior cruciate ligament reconstruction (MOCHA Trial): study protocol for a double-blind, randomized, placebo-controlled clinical trial

**DOI:** 10.1186/s13063-021-05982-3

**Published:** 2022-01-31

**Authors:** Cale A. Jacobs, Caitlin E. W. Conley, Virginia Byers Kraus, Drew A. Lansdown, Brian C. Lau, Xiaojuan Li, Sharmila Majumdar, Kurt P. Spindler, Nicole G. Lemaster, Austin V. Stone

**Affiliations:** 1 University of Kentucky, 740 S Limestone, Suite K401, Lexington, Kentucky 40536-0284 USA; 2 Duke University, Durham, USA; 3 University of California, San Francisco, San Francisco, USA; 4 Cleveland Clinic Foundation, Cleveland, USA

**Keywords:** Anterior cruciate ligament, Montelukast, Posttraumatic osteoarthritis, MRI, Biomarker

## Abstract

**Background:**

After anterior cruciate ligament (ACL) reconstruction, patient-reported outcomes are improved 10 years post-surgery; however, cytokine concentrations remain elevated years after surgery with over 80% of those with combined ACL and meniscus injuries having posttraumatic osteoarthritis (PTOA) within 10–15 years. The purpose of this multicenter, randomized, placebo-controlled trial is to assess whether a 6-month course of oral montelukast after ACL reconstruction reduces systemic markers of inflammation and biochemical and imaging biomarkers of cartilage degradation.

**Methods:**

We will enroll 30 individuals undergoing primary ACL reconstruction to participate in this IRB-approved multicenter clinical trial. This trial will target those at greatest risk of a more rapid PTOA onset (age range 25–50 with concomitant meniscus injury). Patients will be randomly assigned to a group instructed to take 10 mg of montelukast daily for 6 months following ACL reconstruction or placebo. Patients will be assessed prior to surgery and 1, 6, and 12 months following surgery. To determine if montelukast alters systemic inflammation following surgery, we will compare systemic concentrations of prostaglandin E2, monocyte chemoattractant protein-1, and pro-inflammatory cytokines between groups. We will also compare degradative changes on magnetic resonance imaging (MRI) collected 1 and 12 months following surgery between groups with reductions in early biomarkers of cartilage degradation assessed with urinary biomarkers of type II collagen breakdown and bony remodeling.

**Discussion:**

There is a complex interplay between the pro-inflammatory intra-articular environment, underlying bone remodeling, and progressive cartilage degradation. PTOA affects multiple tissues and appears to be more similar to rheumatoid arthritis than osteoarthritis with respect to inflammation. There is currently no treatment to delay or prevent PTOA after ACL injury. Since there is a larger and more persistent inflammatory response after ACL reconstruction than the initial insult of injury, treatment may need to be initiated after surgery, sustained over a period of time, and target multiple mechanisms in order to successfully alter the disease process. This study will assess whether a 6-month postoperative course of oral montelukast affects multiple PTOA mechanisms. Because montelukast administration can be safely sustained for long durations and offers a low-cost treatment option, should it be proven effective in the current trial, these results can be immediately incorporated into clinical practice.

**Trial registration:**

ClinicalTrials.govNCT04572256. Registered on October 1, 2020.

**Supplementary Information:**

The online version contains supplementary material available at 10.1186/s13063-021-05982-3.

## Background

After anterior cruciate ligament (ACL) reconstruction, patient-reported outcomes are improved 10 years post-surgery [1]. Cytokine concentrations, however, remain elevated years after surgery with over 80% of patients with combined ACL and meniscus injuries developing posttraumatic osteoarthritis (PTOA) within 10–15 years after injury [2, 3]. Since pain nociceptors are not located in the articular cartilage, patient-reported outcomes improve despite progressive, irreversible cartilage loss, thus making PTOA a “silent killer” [4]. Because early cartilage loss progresses without pain and dysfunction, the prevalence of PTOA continues to increase. PTOA now represents the most common cause of military disability [5, 6] and is increasingly more common in the general population with more than 5.6 million Americans suffering from PTOA [7, 8].

Our recent results illustrate the downstream cytokine and degradative enzyme activity following ACL reconstruction. ACL and meniscus injury initiate a biochemical cascade resulting in cartilage degradation [9–11], and this process involves an upregulated pro-inflammatory response with a dysregulated anti-inflammatory response [12, 13]. Single-dose intra-articular anti-inflammatory treatment has been reported to reduce hyaline cartilage degradation shortly after the time of injury based on synovial fluid measures of type II collagen degradation [4]. The intra-articular inflammatory milieu at the time of surgery appears to predict the patient symptom state 2 years later [14]; however, the effectiveness of preoperative anti-inflammatory treatments in impacting patient symptoms or slowing long-term PTOA progression is as yet unclear. A lack of efficacy in preoperative interventions may be attributed to a profound inflammatory stimulus from surgical reconstruction of the ACL. The postoperative inflammatory cascade results in articular cartilage and meniscus degradation due to matrix degrading enzymes, especially those which breakdown type II collagen [15].

PTOA affects the whole joint organ including the cartilage, synovium, and bone. PTOA progression is multifaceted and includes activation of the pro-inflammatory NFκB pathway, an increase in pro-inflammatory M1 macrophages, cell senescence, and bone remodeling [16–20]. Limiting the biochemical cascade through an innovative disease-modifying treatment to target upstream activity will potentially treat all components of the knee, thereby lessening the inflammatory response, reducing cartilage catabolism, and potentially improving pathologic bony remodeling observed after ACL reconstruction. The early proteomic PTOA response is more similar to inflammatory rheumatoid arthritis than idiopathic OA [13]; thus, long-acting agents which better regulate pro-inflammatory cytokine activity may more successfully limit tissue destruction [21]. By repurposing approved therapeutics with proven immune efficacy [22], we may arrive at a readily available and cost-effective strategy for disease modification.

Montelukast was first approved for clinical use in 1998 for prophylaxis and chronic treatment of asthma. The drug selectively inhibits the cysteinyl leukotriene CysLT1 receptor. Montelukast blocks the actions of cysteinyl leukotriene LTD4, which is produced through the arachidonic acid pathway. This pro-inflammatory signal is released from several cells including the inflammatory mast cells and eosinophils [23].

Montelukast may also address multiple PTOA mechanisms by inhibiting cysteinyl leukotrienes. Cysteinyl leukotriene inhibition in animal and laboratory models of PTOA resulted in the elimination of senescent cells, reduced NFκB activation, and decreased concentrations of pro-inflammatory and catabolic factors and reactive oxygen species (ROS) while increasing expression of anti-inflammatory factors (inducing anti-inflammatory M2 macrophage infiltration), inhibiting osteoclastogenesis, and improving bone quality [24–29]. The novel use of oral montelukast offers the potential of a disease-modifying treatment to prevent irreversible cartilage loss after ACL injury. The purpose of this multicenter randomized, placebo-controlled trial is to assess whether a 6-month course of oral montelukast after ACL reconstruction reduces systemic markers of inflammation and biochemical and imaging biomarkers of cartilage degradation. Our overarching hypothesis is that a 6-month postoperative course of oral montelukast will reduce PTOA progression after ACL reconstruction by inhibiting multiple targets.

## Methods/design

### Aims

The primary aim of this trial is to determine if a 6-month course of daily oral montelukast alters the biochemical mechanisms of PTOA when compared to placebo. The secondary aim is to assess the effect of oral montelukast on cartilage degradation and bone remodeling after ACL reconstruction. The hypotheses for the primary and secondary aims are that the use of montelukast after ACL reconstruction will:

- Reduce peripheral concentrations of prostaglandin E2 (PGE2), monocyte chemoattractant protein-1 (MCP-1), and pro-inflammatory cytokines.
- Reduce degradative changes on magnetic resonance imaging (MRI) at 1 year, in association with reductions in early biomarkers of cartilage degradation (C-terminal crosslinked telopeptide type II collagen (CTXII)) and bony remodeling (an alpha isomerized version of the C-terminal crosslinked telopeptide of type I collagen (CTXIα)).

The complimentary aims will allow assessment of the joint as an organ and provide evidence for a novel, disease-modifying treatment approach with an approved, readily available, and widely used therapeutic agent to target multiple mechanisms of PTOA.

### Participants

Participants will include 30 individuals undergoing primary ACL reconstruction (15 in the treatment group and 15 in the placebo group). Potential study participants will be identified by members of the research team during regularly scheduled office visits at the University of Kentucky’s Orthopaedic & Sports Medicine or University of California San Francisco Sports Medicine Center. After being identified as a potential study participant, the patient will meet with a member of the research team coordinator. Provided that the patient meets all inclusion and exclusion criteria, a member of the research team will begin the process to obtain informed consent. As part of the consent process, patients will have the option to allow the investigative team to store their data and biological specimens for future ancillary studies. We routinely recruit subjects from these physicians’ practices using similar methods.

In March, 2020, the FDA required a Boxed Warning about serious mental health side effects for montelukast which includes the risks of suicidal behavior and other serious neuropsychological events [30]. Children and adolescents will be excluded from the current trial as neuropsychological side effects of montelukast have been reported to be more common in children and adolescents than adults [31, 32]. To further avoid the potential for exacerbation of a pre-existing psychological condition, we will also exclude those with depressive symptoms and those that endorse suicidal ideation at the time of enrollment. Psychological screening and safety monitoring will be described in more detail below.

This trial will specifically target those that may be at greatest risk of a more rapid PTOA onset (age range 25–50 with concomitant meniscus injury as evidenced on their clinical preoperative MRI and clinical exam) [33–35]. Those who are undergoing revision procedures will be excluded; however, patients will not be excluded if they have had a previous contralateral ACL injury and/or surgery. In doing so, we hope to address the needs of the most at-risk patients but also those in whom a treatment signal could be feasibly detected.

#### Age ≥ 25–50

The response to injury may differ between younger and relatively older patients. In a large cohort study, increased age at the time of surgery was identified as an independent risk factor for radiographic PTOA [33, 34]. Additionally, the initial insult of ACL injury is commonly associated with subchondral bone marrow lesions [36]. While midterm outcomes for younger patients do not appear to be impacted by the presence of bone marrow lesions, older patients have demonstrated self-reported pain, functional limitations, and greater cartilage degradation [19, 37, 38]. Patients > 25 years of age may then display worse symptoms and more rapid PTOA progression thereby potentially providing a signal that may be more readily monitored when compared to younger patients. For these reasons, we have elected to enroll individuals between 25 and 50 years of age.

#### Concomitant meniscus injury

Similar to increased age, the presence of meniscus injury was identified as an independent risk factor for radiographic PTOA in a large cohort study [33, 34]. The role of the meniscus injury on PTOA progression may be related to the persistent intra-articular inflammatory response seen after ACL reconstruction [2, 15]. We have demonstrated that pro-inflammatory stimulation of meniscus cells increases matrix metalloproteinase and cytokine activity [10, 15, 39]; the combination of pro-inflammatory cytokines and compressive loading, comparable to sporting and high demand activities, results in further degradative enzyme activity and increased production of pro-inflammatory mediators [40]. In this way, the meniscus plays an active role in promoting the cycle of articular cartilage degradation and PTOA progression after ACL reconstruction. We therefore opted to specifically target patients with concomitant meniscus injury. We will include patients with either medial or lateral meniscus tears evident on preoperative MRI, but should an enrolled participant be found to not have a meniscus tear at the time of surgery, the participant will not be randomized and will be removed from the study. The size, location, and type of meniscal tear will be documented at the time of surgery as will details related to the surgical treatment (repair vs. meniscectomy).

### Design and setting

**Fig. 1:**
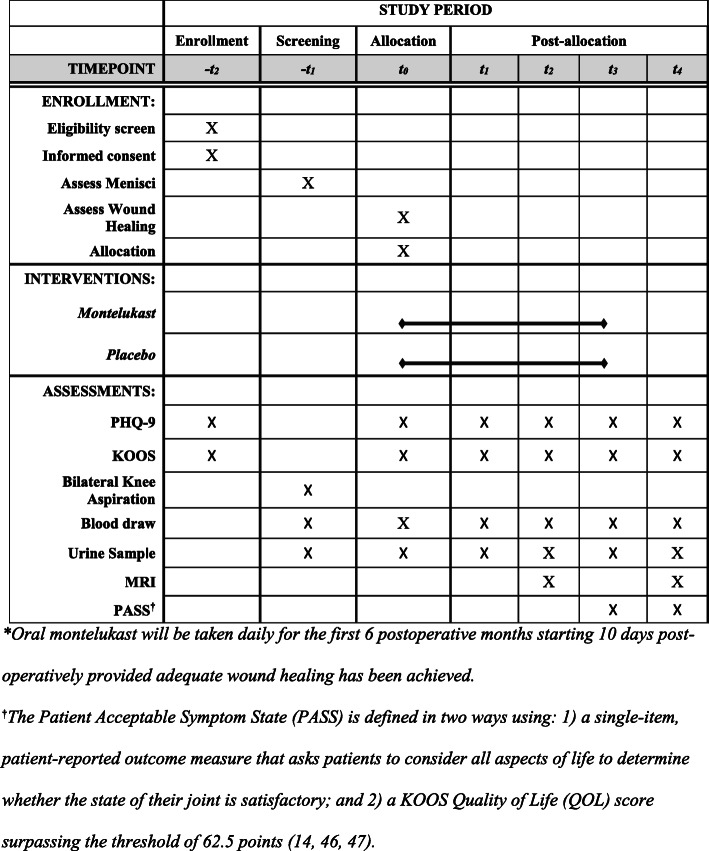
Schedule of enrollment, interventions, and assessments

This is a multicenter, superiority RCT with parallel group assignment and 1:1 allocation ratio to assess the use of montelukast after ACL reconstruction. The study has been approved by both the University of Kentucky and University of California San Francisco institutional review boards. Details of the study design are set out in Fig. 1 according to the Standard Protocol Items: Recommendations for Interventional Trials (SPIRIT) Statement [41]. The SPIRIT Checklist can be found in Additional file 1. Participants will be enrolled at two academic medical centers (University of Kentucky and University of California San Francisco) with study-related analyses being performed at the Cleveland Clinic and Duke University. Important protocol modifications will be communicated to the investigative team through regular weekly meetings. It will be the PI’s responsibility to update participants or trial registries on any protocol modifications.

### Intervention

Patients aged 25 to 50 years will randomly be assigned to receive one daily oral montelukast (10 mg) versus oral placebo daily for 6 months after surgery. Montelukast tablets will be encapsulated in in blue and white gelatin capsules (F05280-1000, Capsuline, Dania Beach, FL) with corn starch filler used to fill the remaining space (Batch/Lot number 176537, Medisca, Plattsburgh, NY). The oral placebo will be the same cellulose capsule with only starch filler. Both the study drug and placebo will be prepared by each institution’s Investigational Drug Service. An oral placebo was selected to offset the potential perceived advantages of daily oral drug delivery ensuring that any treatment effect would be due to the study drug and not differences in drug appearance, mode of delivery, and/or dosing frequency and duration. Prostaglandins and leukotrienes mediate inflammation and are produced through the metabolism of arachidonic acid; blocking the production of these mediators has been shown to have potential anti-inflammatory and chondroprotective effects [42, 43]. Oral montelukast is a selective leukotriene receptor antagonist and, as previously described, induces anti-inflammatory M2 macrophage infiltration, inhibiting osteoclastogenesis and improving bone quality [24–29].

In addition to the aforementioned anti-inflammatory and chondroprotective effects, montelukast was selected based on 3 other key criteria: (1) oral administration, (2) postoperative timing of use, and (3) sustained method of delivery.

#### Oral administration

In our recent experience with studies involving intra-articular injections with ACL-injured patients (NCT02930122), we have observed that a relatively high percentage of eligible subjects (31%) chose not to participate in the studies specifically because they would be undergoing an intra-articular injection and/or having a “fear of needles.” Oral montelukast may overcome or lessen this barrier as it has been reported to be preferred by asthmatic patients and thereby engenders greater compliance when compared to inhalants [44]. The current study will allow us to assess the feasibility and acceptability of montelukast compared to our previous experiences with intra-articular agents (NCT02930122) [4, 15].

#### Postoperative administration

The ability to initiate montelukast 10 days after surgery offers multiple advantages. First, we have previously described the logistical challenges to enroll patients within 8 days of injury in order to administer intra-articular anti-inflammatory injections [9]. Perhaps more important than early preoperative patient access is the ensuing surgical insult to the joint. While we have demonstrated that preoperative corticosteroid injection reduces biomarkers of cartilage degradation [4], the surgery reinitiates a secondary inflammatory response [15]. Further, this inflammatory response after ACL reconstruction was both larger in magnitude and duration when compared to the initial ACL rupture [15]. Intra-articular concentrations of pro-inflammatory cytokines, interleukin-6 (IL-6), and IL-1β, were nearly twofold greater 1 week post-surgery than 1 week after the initial injury. IL-6 and IL-1β were remained significantly elevated 4 weeks following ACL reconstruction when compared to concentrations assessed on the day of surgery [15]. These results suggest a potential need for postoperative treatments that may be of critical importance to alter the biochemical joint catabolic cascade induced by surgery that likely contributes to progressive cartilage degradation and PTOA. Moreover, it may be necessary to alleviate the overwhelming biochemical surgical insult before it is possible to discern any real benefits of early post-injury interventions.

#### Sustained delivery

We have previously reported that the proteomic changes after ACL injury are more similar to changes associated with rheumatoid arthritis than idiopathic osteoarthritis [13]. This response is not only reinitiated after surgery, but is maintained perhaps indefinitely as increased synovial concentrations of pro-inflammatory cytokines have been reported 5 years after ACL reconstruction [2]. It has been previously suggested that long-acting agents may provide a greater treatment effect as temporal regulation of cytokine activity may more successfully alter the pro-inflammatory environment than shorter-duration treatments [21]. Prior studies have shown that montelukast can counteract the inflammatory environment of rheumatoid arthritis [45]. As such, similar to the treatment of rheumatoid arthritis, a sustained treatment may be necessary to modify the PTOA disease process, providing additional justification to test a 6-month course of oral montelukast after ACL reconstruction.

#### Safety monitoring

A safety review of treatment group will be performed with safety endpoints being monitored at each study visit. Due to an FDA black box warning related to montelukast, we will utilize the Patient Health Questionnaire-9 (PHQ-9) to quantify depressive symptoms and suicidality at all clinic-based study visits. The PHQ-9 is a validated, self-administered screening instrument that asks patients to select how often they have been bothered by specific symptoms in the last 2 weeks [46, 47]. The PHQ-9 consists of 9 questions that parallel the diagnostic criteria for Major Depression Disorder from the Diagnostic and Statistical Manual of Mental Disorders. Each item is scored from 0 to 3 with 3 representing more severe symptoms. PHQ-9 scores range from 0 to 27, and scores of 15 or greater are indicative of severe depression [46]. After providing informed consent, patients will complete a baseline PHQ-9 questionnaire. Should the subject have a PHQ-9 ≥ 15 at baseline, they will be withdrawn from the study. If the patient is not currently being observed and/or treated for depression, they will be then referred to their primary care physician or mental health professional for evaluation. The PHQ-9 also has a question that is specific to suicidal thoughts. Should the subject endorse suicidal ideation at baseline, the subject will be withdrawn from the study prior to initiating treatment and a “warm hand-off” will be performed and a risk assessment will be performed by the local psychologist that is a part of the study team.

##### Study stopping rules

Neuropsychiatric symptom severity and suicidality will be monitored closely within study procedures and reviewed weekly by the study’s medical officer and the principle investigator, and referrals for appropriate levels of care will be done as needed. At all study postoperative follow-up visits, subjects will complete the PHQ-9. Similar to the enrollment screening procedures, treatment will be immediately stopped for subjects with scores ≥ 15 or if they endorse suicidal ideation. The subject will then be referred to their primary care physician, mental health professional, or the study’s local psychologist that is a part of the study team as described above. In addition, consistent with the FDA’s guidance [30], treatment will also be immediately stopped if patients experience a change or increase in any of the following behavior or mood-related changes:

**Table:** 

Agitation, including aggressive behavior or hostility	Obsessive-compulsive symptoms
Attention problems	Restlessness
Bad or vivid dreams	Sleepwalking
Depression	Stuttering
Disorientation or confusion	Suicidal thoughts and actions
Feeling anxious	Tremor or shakiness
Hallucinations	Trouble sleeping
Irritability	Uncontrolled muscle movements
Memory problems	

In addition, while to our knowledge there have been no human reports of impaired wound healing associated with montelukast use, there have been studies utilizing different animal models that have reported the potential for delayed healing. The animal studies that reported impaired healing or decreased angiogenesis used doses of 10 mg/kg/day [48–50] whereas other studies reported improved healing characteristics at either the same dose (10 mg/kg/day, [51, 52]) or lower dose (3 mg/kg, [53]). In the one study that assessed the effects on human tendon fibroblasts, the effect did appear to be dose dependent with an increased effect on fibroblasts with increasing doses [54]. We do not expect wound complications since our study will be using a dosage of approximately 0.14 mg/kg/day (10 mg/day assuming an estimated body mass of 70 kg), and there have been no clinical cases reported to date; however, we will closely monitor the surgical incisions for signs of delayed healing at all routine standard of care visits in addition to the 1-month study visit. Furthermore, postoperative montelukast will not be initiated until 10 days following surgery once there is evidence of sufficient wound healing. This is the time frame during which patients undergoing ACL reconstruction routinely have their sutures removed and begin to shower without having the wound covered. Should a patient not have adequate wound healing at the 10-day time point, they will not be randomized and will be withdrawn from the study.

Breaking the randomization code will be performed by the study’s medical officer and will occur if it is necessary for the care of the subjects. For example, if an adverse event or serious adverse event occurs with a subject and knowledge of the drug randomization would be important for clinical care or to determine appropriate treatment, then the principal investigator will break the code and the subject will be withdrawn from the study. If a subject discontinues the study for any reason prior to the 6-month follow-up visit, then the collected data will be evaluated but not included in the analysis and a replacement subject will be enrolled in the study. There are no provisions for ancillary or post-trial care.

#### Blinding procedures

The principal investigator, patients, and assessors will be blinded to group assignment. Using broad and previously established enrollment criteria, we hope to reduce selection bias to a minimum while protecting potentially vulnerable individuals through the exclusion criteria. Procedural bias and measurement bias will be reduced by the blinded data analysis.

##### Randomization

Following informed consent and after successfully being screened into the study based on intraoperative findings and adequate wound healing 10 days following surgery, subjects will be randomized to one of two groups. The randomization schedule will be made by a predesignated unblinded study team member that will not be involved in data collection or analysis. Using a random number generator, allocation of drug or placebo will be by permutated random block size, ensuring that approximately equal numbers of subjects will be treated with drug or placebo in the unlikely event that the study will need to be terminated prematurely. Should a subject discontinue the study for any reason prior to 6-month follow-up visit, then the collected data will be evaluated but not included in the analysis and a replacement subject will be enrolled in the study. As such, the randomization schedule will be prepared for 40 potential participants despite having a target enrollment of 30 participants to ensure adequate power.

##### Blinding

The team member that prepared the randomization schedule will meet with the pre-specified unblinded study personnel prior to the site initiation visit. The unblinded personnel will then prepare sealed envelopes that contain each subject’s group assignment.

We will designate, a priori, study personnel that will be unblinded. These unblinded personnel will be directly involved with preparing the blinded medications and maintaining the drug log. The unblinded pharmacist/coordinator will access the actual treatment assignment but both blinded study personnel and the patient will not know the patient’s treatment assignment. Treatments will be prepared by designated unblinded personnel who will have no other patient contact.

Should an investigator become inadvertently unblinded, the principal investigator and medical officer will determine the extent of the unblinding and potential ramifications. If necessary, the investigator’s role may need to be altered to only include tasks affiliated with unblinded personnel. Should a patient become inadvertently unblinded, that patient’s data will continue to be collected for safety purposes; however, the data will not be used for the final analyses and a replacement subject will be enrolled. Unblinding of a subject(s) group assignment will be documented and will include an explanation of why the study medication was unblinded. Following data lock, group assignment can be unblinded and the unblinded information can be sent to the study participants at the discretion of the principal investigator.

#### Adherence

Adherence to the treatment protocol will be assessed and verified through the completion of the drug log. The drug log will be maintained by unblinded study personnel and inspected on no less than an annual basis by an unblinded study co-investigator.

Patient adherence to the medication will be documented through weekly patient self-report. The patients will be asked to take 1 pill daily during the 6-month prescription, thus 1 day will equate to 1 pill. Patients will be provided a weekly SMS text notification with a link to REDCap to ask if the patient has taken the medication that day. Previous literature has defined good medication compliance as taking at least 80% of the prescribed medication [55]. We will calculate the proportion of patients who meet this criterion in the treatment group as well as the 95% confidence interval (CI) around this compliance rate. The treatments will be considered feasible and acceptable if over 70% of participants reach this threshold.

#### Minimizing attrition

In order to maximize retention participants will be asked to provide alternate contacts and to specify whether study staff may leave messages or send mail. To promote attendance, patients will be contacted by phone both 1 week and 1 day prior to their study visits. In addition to ease the burden of attending subject visits they will be compensated for attending the follow-up study visits. The research coordinator and the PIs will track all study visits and will follow-up on any missed visits. Proactive strategies will be developed to work with participants who have trouble attending study visits. Participants will have 7:30 am–5 pm access to research personnel. In addition, while there are several study visits, all study visits can be scheduled to coincide with their physician follow-up appointments. We are currently employing these strategies in several ongoing trials and we have consistently resulted in less than 10% of ACL-reconstructed patients being lost to follow-up from our previous and ongoing clinical trials.

#### Confidentiality

Once enrolled, patients will be identified with a study ID in all study documentation, both written and in REDCap. Identifiable information (name, medical record number) will be kept separate from all study data and will be located on the password-protect excel spreadsheet on the secure department server behind the UK HealthCare firewall. The password will only be known by appropriate study personnel that are included in the approved IRB Study Personnel list. The identifiable information will not be transferred or collected in the data collection forms used for the study.

#### Data monitoring

Clinical site monitoring will be conducted by a member of the research team, Caitlin Conley, PhD, to ensure that the rights and well-being of trial participants are protected, that the reported trial data are accurate, complete, and verifiable, and that the conduct of the trial follows the currently approved protocol/amendment(s), with International Conference on Harmonisation Good Clinical Practice (ICH GCP), and with applicable regulatory requirement(s).

Remote clinical monitoring will be performed within 2 weeks following enrollment of the first subject, and repeated bi-annually (6 months) thereafter. Remote monitoring will include comprehensive, 100% data verification. Independent audits will not be conducted by the study sponsor to ensure monitoring practices are performed consistently across all participating sites as this is a single-site study.

Dr. Conley will conduct monitoring to verify that (a) the rights and well-being of human subjects are protected; (b) the reported trial data are accurate, complete, and verifiable from source documents; (c) the conduct of the trial is in compliance with the currently approved protocol/amendment(s), Food and Drug Administration (FDA) regulations and ICH guidelines; (d) ensure the quality and integrity of clinical study data; and (g) evaluate the progress of the study and ensure completion of the clinical study in a timely manner.

### Data collection

#### MRI analyses

All subjects will undergo MRI examination at visit 4 (1 month after ACL reconstruction) and visit 6 (12 months postoperatively). At one of the enrollment sites, the average time between initial presentation to the orthopedic surgeon and the date of surgery is 9 days. As such, it is often difficult logistically for patients and/or their caregivers to attend a study-specific MRI visit preoperatively when they have already had to take time away from school or work to have the clinical MRI performed in order to confirm the original injury diagnosis. Thus, we will rely on the clinical MRI and clinical exam to determine eligibility and have opted to have the baseline research protocol MRI images collected 1 month postoperatively as this is more feasible for patients and this timing is consistent with other groups studying cartilage changes after ACL reconstruction [56].

At both enrollment sites, sagittal and axial MRI images will be acquired using a 3.0-T MRI scanner and an 1Tx/15Rx phased array knee coil/flex coil. Sagittal and axial T1-weighted fast spin echo images (FSE) will be acquired to allow semiquantitative analysis at the conclusion of the study. These sequences will be followed by a sagittal high-resolution 3D dual echo steady state (DESS) sequence for cartilage segmentation and a sagittal combined T1ρ/T2 mapping for the quantification of cartilage composition. The major parameters for the DESS sequences are as follows: FOV = 140 mm, matrix = 384 × 307 × 176, resolution = 0.36 × 0.45 × 0.7 mm^3^, time of repetition (TR)/time of echo (TE) = 17.6 ms/6.0 ms. The T1ρ/T2 mapping will be acquired using a 3D MAPSS sequence with major parameters: FOV = 140 mm, image matrix = 320 × 160 × 24, resolution = 0.44 × 0.44 × 4 mm^3^, time of recovery = 1.5 s, bandwidth = 400 Hz/Pixel, TR/TE = 7 ms/3 ms, time of spin lock (TSLs) = 0, 10, 40, 80 ms for T1ρ mapping and preparation TEs = 0, 20, 40, 60 ms for T2 mapping. The scan time will be approximately 12 min for the combined T1ρ/T2 mapping. Images will be obtained with the knee in full extension.

Images collected at the University of Kentucky will then be transferred digitally to the University of California, San Francisco (UCSF) to be analyzed in the Majumdar/Link Lab which is part of the Musculoskeletal and Quantitative Imaging Research Group (MQIR) at UCSF. Based on the high-resolution DESS images, articular cartilage will be segmented using a spline-based, semiautomatic technique. The T1ρ map will be created on a voxel-by-voxel basis using established fitting routines. The segmented masks will then be overlaid on the T1ρ maps after registration between DESS and T1ρ images, and mean T1ρ values will be calculated for the entire tibiofemoral cartilage, medial and lateral tibial plateau, medial and lateral femoral condyles, and the patellofemoral compartment will be calculated. Semiquantitative analyses will also be performed to assess abnormalities of the cartilage, meniscus, and bone marrow by an experienced musculoskeletal radiologist using the MRI Osteoarthritis Knee Score (MOAKS) [57].

In addition to assessing cartilage changes on MRI, we will also assess bone shape changes after ACL reconstruction. The bone area of the medial femoral condyle has been shown to increase within the first few months after ACL reconstruction [58, 59]. This is clinically meaningful as similar bony changes have been previously associated with progressive OA and within 6 months of ACL reconstruction correlated with subsequent patient-reported outcomes and MRI markers of cartilage quality at 3 years [58, 59]. Dr. Li’s group at the Cleveland Clinic has previously established the methods to assess bone shape changes after ACL reconstruction [59]; images from both enrollment sites will be transferred digitally to the Cleveland Clinic for analysis.

#### Patient-reported outcomes

In addition to the aforementioned assessments of depressive symptoms with the PHQ-9 questionnaire, we will also be assessing knee-related patient-reported outcomes at the five clinic-based study visits using the Knee injury and Osteoarthritis Outcome Score (KOOS). The KOOS is comprised of 5 subscales (Pain, Symptoms, Activities of Daily Living, Sports and Recreation, and Quality of Life) with each scale being scored from 0 to 100 with higher scores being indicative of a superior outcome [60]. In addition, we will calculate the KOOSglobal score, which is a global score indicative of overall knee symptoms and function that has been validated in ACL reconstruction patients [61], as well as the patient acceptable symptom state (PASS) from the KOOS quality of life domain [14, 62].

At the 6- and 12-month follow-up visits, participants will also be asked to complete the single patient acceptable symptom state (PASS) question, which requires the participant to consider all aspects of life to determine if the state of the knee is “satisfactory” 1 year following surgery and postoperative treatment [63]. The specific phrasing of the PASS question will be, “Taking into account all the activity you have during your daily life, your level of pain, and also your activity limitations and participation restrictions, do you consider the current state of your knee satisfactory?” to which the participants will have the option to respond either “yes” or “no” [63].

#### Biomarker analyses

Blood and urine specimens will be collected on the day of surgery and all postoperative study visits to determine concentrations of the following biomarkers of inflammation, NFκB activation, cartilage degradation, and bone turnover. The selected urinary biomarkers have been shown to be predictive of inferior clinical outcomes and cartilage thinning [14, 64]. These validated biomarkers allow accurate assessment of cartilage degradation and bony remodeling and will be performed at the Duke Molecular Physiology Institute’s Biomarkers Shared Resource. In addition, both the involved and contralateral knees will be aspirated and synovial fluid collected on the day of surgery to assess inflammatory mediators and biomarkers of cartilage degradation. To avoid dry aspirations and missing data, the knee aspirations will be performed with a 5-mL saline lavage and normalized to urea content [65]. We anticipate that increased synovial fluid concentrations of a biomarker of proteoglycan loss (glycosaminoglycan (GAG)) and inflammatory mediators at the time of surgery would be predictive of increased MRI evidence of cartilage degradation postoperatively for the control group but would not be associated with increased cartilage changes in the montelukast group should that treatment be effective.

##### Inflammatory markers

Enzyme-linked immunosorbent assays (ELISAs) will be used to measure concentrations of IL-1β, IL-2, IL-6, IL-8, TNFα, IFN-γ, IL-10, IL-12p70, IL-13, and IL-4 (multiplex Proinflammatory Panel 1, Meso Scale Discovery, Rockville, MD) [66], IL-1α (Meso Scale Discovery, Rockville, MD) [4, 9, 14], prostaglandin E2 (PGE2; R&D Systems, Minneapolis, MN) [67], and monocyte chemoattractant protein-1 (MCP-1; R&D Systems, Minneapolis, MN) [68] in both the serum and synovial fluid.

##### Cartilage remodeling

Following ACL injury, proteoglycan loss progresses as evidenced by a decrease in synovial fluid GAG [69]. This loss in proteoglycan content has been reported to be followed by a subsequent onset of collagen damage and an increase in the synovial fluid concentration of biomarkers of type II collagen breakdown (CTXII) with CTXII released into the synovial fluid and the circulation [69]. Urinary CTXII has been identified as a biomarker for the diagnosis, staging, and evaluation of the prognosis of hip and knee OA [70, 71] and has also been demonstrated to be responsive over short testing periods (3 months) [72]. We have reported that CTXII correlates with the degree of joint destruction and increases significantly within 1 month after ACL injury [4, 9]. In addition to markers of cartilage degradation, we will also assess a biomarker of type II collagen synthesis in the serum and synovial fluid (N-terminal propeptide of collagen IIA (PIIANP) as low-serum PIIANP has been predictive of clinically relevant progression of both OA symptoms and cartilage degradation [64].

Synovial fluid concentrations of GAG will be quantified using a commercial kit per the manufacturer’s instructions (Kamiya Biomedical Company, Seattle, WA, USA) as previously described [69]. CTXII will be measured in the urine by ELISA (Cartilaps® (CTX-II); Immunodiagnostic Systems, Inc, Fountain Hills, AZ) [4, 9] and will be normalized to creatinine levels (Quidel, San Diego, CA) [64, 73]. PIIANP will be measured in the serum and synovial fluid by ELISA (Merck Group/Millipore, EZPIIANP-53 K, [64]).

##### Bony remodeling

CTXIα is localized to areas of high turnover of subchondral bone [73] and has been found to be predictive of OA symptom and radiographic progression [64]. CTXIα will be measured in the urine by sandwich ELISA (Nordic Biosciences, Herlev, Denmark), and like CTXII, will be normalized to urinary creatinine levels [64, 73].

#### Rigor and reproducibility

Biomarker data collected will be analyzed with respect to variability, linear range of standard, and need for repeat analyses. Controls provided with commercially available ELISA kits will be used with every run. For assays for which no control is available or provided, aliquots of serum from normal human subjects have been aliquoted and frozen at – 80 °C for this purpose. Each assay day, a fresh aliquot of this control serum is thawed and used on every plate to calculate intra- and inter-assay variance of the assay. In addition to the standard curve run in duplicate for each assay, this control will be run with each assay and the results used to determine the precision of the assay and to establish an acceptable control range for the assay. The mean of the control sample for all assays plus or minus two standard deviations is defined as the acceptable control range. Any samples on a plate in which the control falls outside of this range will be excluded and repeated. Samples will be run in duplicate and reanalyzed if the coefficient of variation is greater than 15%. For values that are below the level of detection as defined by the lowest standard, a value equivalent to 0.5 lowest limit of detection will be recorded and used for statistical analyses. To ensure data quality and fidelity, all data collection and analysis procedures will be detailed in a structured manual. The full research team will attend half-day training sessions both prior to study initiation and following enrollment of the first three patients have been enrolled to identify unforeseen issues that may arise and will also convene quarterly throughout the study period.

For cartilage T_1ρ_ and T_2_ quantification, we have published excellent scan/re-scan reproducibility in human subjects with coefficients of variation (CV) < 4% for single vendor settings [74] and CV < 10% for multi-vendor settings [75] using the MAPSS sequences [76]. Longitudinal CVs of monthly collected phantom T_1ρ_ and T_2_ have been reported < 3% [74]. In this study, a T_1ρ_ and T_2_ phantom will be scanned monthly at each site for the duration of the proposed study. SNR, intra-day reproducibility, and inter-day reproducibility will be quantified. Longitudinal data will be examined for any potential shift of quantified parameters. If more than 3% of relaxation time shift is observed, acquisition of subjects will be put on hold until discrepancies can be resolved.

### Statistical analysis

#### Primary aim analysis

Inflammatory biomarkers PGE2 and MCP-1 concentrations will be assessed for normality [12, 66]. If not normally distributed, biomarker concentrations will be transformed using Box-Cox transformation [66]. A 2 × 4 repeated measures ANOVA (group × time) will be used to assess changes in biomarker concentrations between the montelukast and placebo groups.

#### Secondary aim analysis

Variables will be assessed for normality and if normally distributed, baseline and 1-year T1ρ and T2 relaxation times as well as bone shape changes will be compared between groups using a 2 × 2 repeated measures ANOVA (group × time). Based on previous work, it is expected that the biomarkers of type II cartilage breakdown (CTXII) and bony remodeling (CTXIα) will not be normally distributed and it is anticipated that biomarker concentrations will be transformed using Box-Cox transformation [66]. A 2 × 4 repeated measures ANOVA (group × time) will be used to assess changes in biomarker concentrations between the montelukast and placebo groups. The ordinal semiquantitative MOAKS data will be compared between groups using chi-square or Fisher exact tests as appropriate.

For both primary and secondary analyses, in the case of participants that violate group allocation, we will perform Intention-to-Treat and Per Protocol analyses. We will not employ imputation methods in the case of missing data. No interim or other subgroup analyses will be performed.

#### Relevant covariates

Covariates known to influence the selected biomarkers will be collected and included as covariates in the statistical analyses which include patient age, biological sex, and race [77]. The location of significant differences will be determined using post hoc tests with a Bonferroni correction.

#### Power analysis

The primary outcome variable is the change in medial femoral condyle T1ρ relaxation time on MRI between 1 and 12 months postoperatively. Based on a previous study of ACL-reconstructed patients, we assume a standard deviation of 2.86 ms [78]. With a sample size of 15 patients per group, the trial is 81.5% powered to detect differences of 3.09 ms between groups (effect size = 1.08). A difference of this magnitude is feasible when one considers the differences previously noted for ACL reconstructed knees with concomitant meniscus tears [78]. This sample size is feasible for this multicenter trial. In a review of all ACL-injured patients seen at the University of Kentucky in 2019 (Cale Jacobs, unpublished raw data), 50 patients that met the study inclusion and exclusion criteria were seen. We anticipate a similar volume at the University of California San Francisco thereby providing a recruitment pool of approximately 100 eligible patients per year.

#### Dissemination of results

The investigators will disseminate the results a number of ways. First, the abstract containing the results will be submitted to national or international conference such as the Osteoarthritis Research Society International (OARSI), Orthopaedic Research Society (ORS), or the American Orthopaedic Society for Sports Medicine (AOSSM). Second, the manuscript with these results will be submitted to a peer-review journal for publication. All co-authors included on this manuscript will be included on subsequent works, and additional authors may be added per the group’s discretion based on the contributions to the study and manuscript. No professional writers were used during the preparation of the protocol paper nor will they be used during the preparation of the final manuscript. Third, results will also be reported at ClinicalTrials.gov to allow free access for other research subjects and other parties.

## Discussion

In addition to evaluating the efficacy of postoperative montelukast, we will also be able to assess the feasibility and acceptability of this treatment approach. There are facets to this treatment approach that maximize the opportunity to alter the progression of PTOA but there are also aspects that pose a challenge in the clinical setting. Perhaps the greatest challenge in the treatment of PTOA is that the initial progressive proteoglycan loss and cartilage degradation during the first several years after ACL reconstruction often occurs with little to no worsening of patient symptoms. In fact, when compared to a person’s preoperative state, patient-reported outcomes remain significantly improved at 1, 2, 6, and 10 years after surgery [1, 79]. Progressive, irreversible joint changes occurring without a marked increase in pain or loss of function perhaps poses the greatest challenge in terms of patient compliance or adherence with a 6-month course of montelukast. Patient knowledge has been previously identified as a barrier to compliance. While more than 80% of patients with combined ACL and meniscus injuries have PTOA within 10–15 years after injury [3], patients remain largely unaware of the long-term implications of injury. When asked about expectations prior to ACL reconstruction, 98% of ACL-injured patients expect no risk or a slight risk of osteoarthritic changes within 10 years after surgery [80]. The stark contradiction between the risk of PTOA and patient knowledge of the risk of PTOA after ACL injury, combined with a lack of pain or functional limitation after ACL reconstruction may reduce a patient’s motivation to adhere to the prescribed montelukast treatment plan. To mitigate the risk of non-compliance associated with a lack of knowledge, participants will be given a one-page educational infographic developed by the Osteoarthritis Action Alliance (www.oaaction.unc.edu) that highlights the prevalence of PTOA after ACL reconstruction. This form also addresses rehabilitation, diet, and exercise considerations which may offset the risk of OA progression. The infographic is not be specific to the study and does not contain information about the use of montelukast as a potential treatment option. As such, the publicly available infographic may be provided to all patients undergoing ACL reconstruction regardless of study participation.

In addition to patients generally lacking knowledge of the risk of PTOA after ACL injury, there are other factors that may influence patient adherence to postoperative montelukast treatment. The ACL population is generally younger, and age < 40 years has been previously associated with poorer compliance with medication [81]. Also, compliance is reduced when side effects are experienced. For example, in a randomized, placebo-controlled trial that included 232 OA patients assessing the efficacy of doxycycline, of the 28 patients prematurely discontinued treatment, the vast majority (24/28) cited adverse events as the underlying reason [55]. Montelukast has been widely used as a treatment for seasonal allergies, with more than 9.3 million Americans being prescribed montelukast in 2018 alone [30]. However, all medications carry a risk of side effects. As previously described, montelukast has a risk of neuropsychiatric side effects and study participants will be closely monitored. Additionally, a limited number of mixed reports provide conflicting evidence of impaired wound healing associated with montelukast use in animal models [48–54]. To minimize this risk, study participants will only initiate treatment once sufficient healing of the surgical incision has occurred.

Montelukast treatment offers several potential benefits that may promote increased compliance. Oral medications are often more preferable for patients, and the simplicity of taking a single  oral tablet once daily may also increase adherence [82, 83]. Longer duration of treatment can negatively impact adherence but while a 6-month course obviously requires more diligence than a single-dose intra-articular injection, compliance to a 6-month course of medication is greater than durations of 9 months or more [81]. Additionally, 88% of OA patients in the aforementioned doxycycline RCT completed the 6-month course or oral medication [55]. Patients normally resume running 4 to 6 months after surgery as part of a progressive return to sports [84], and patients will be educated of the potential anti-inflammatory benefits this time period. Finally, while the medication will be provided to study participants at no cost, the low cost of oral montelukast may also improve compliance in the clinical setting [81].

The current standard of care for patients with combined ACL and meniscal injuries consists of surgical treatment which successfully restores joint stability; however, this approach does not address the persistent inflammatory process that promotes cartilage degradation and PTOA progression. The current multicenter RCT will assess whether a 6-month postoperative course of oral montelukast affects multiple PTOA mechanisms but also the feasibility and acceptability of the treatment. Because montelukast administration can be administered for extended periods and offers a low-cost treatment option, should it be proven safe and effective in the current trial, these results can inform larger multicenter trials and potentially be immediately incorporated into clinical practice.

## Trial status

This is protocol version 2.0 (September 16, 2020). We anticipate that patient recruitment will begin February 15, 2021, with recruitment ending February 14, 2022.

## Supplementary Information

**Additional file 1.** Standard Protocol Items: Recommendations for Interventional Trials (SPIRIT) Checklist.

## Data Availability

Data will be collected in a protected database with an unidentified ID number provided for each participant. All authors of the current manuscript will have access to the final dataset. Participant-level data are available on request by sending an email to Cale Jacobs. All data will be available for 5 years after the relevant publication.

## Abbreviations

ACL: After anterior cruciate ligament
CI: Confidence interval
CTXII: C-terminal crosslinked telopeptide type II collagen
CTXIα: An alpha isomerized version of the C-terminal crosslinked telopeptide of type I collagen
CV: Coefficient of variation
DESS: Dual echo steady state
ELISAs: Enzyme-linked immunosorbent assays
FOV: Field of view
FSE: Fast spin echo
GAG: Glycosaminoglycan
IFN: Interferon
IL: Interleukin
MAPSS: Magnetization-Prepared Angle-Modulated Partitioned k-space Spoiled Gradient Echo Snapshots
MCP-1: Monocyte chemoattractant protein-1
MOAKS: MRI Osteoarthritis Knee Score
MQIR: Musculoskeletal and Quantitative Imaging Research Group
MRI: Magnetic resonance imaging
OA: Osteoarthritis
PGE2: Prostaglandin E2
PHQ-9: Patient Health Questionnaire-9
PTOA: Posttraumatic osteoarthritis
RCT: Randomized control trial
RA: Rheumatoid arthritis
TNF: Tumor necrosis factor
TE: Time of echo
TR: Time of repetition
TSL: Time of spin lock
